# Treatment of Urethral Stricture

**Published:** 1827-07

**Authors:** 


### TREATMENT OF STRICTURES.

I. *A Treatise on Stricture of the Urethra, &c.* By George Macilwain, Member of the Royal College of Surgeons, &c. 8vo, pp. 138. Anderson.

II. *Practical Observations on the Application of Lunar Caustic to Strictures in the Urethra, &c.* By M. W. Andrews, Member of the Royal College of Surgeons. 2d Ed. pp. 249. Callow and Wilson.

It is not always smooth water and sailing on velvet with surgeons, no more than with physicians. Surgical diseases, although more tangible in their forms, demonstrable in their sites, and traceable as to their causes, than those which are purely medical, are yet so modified by circumstances as to require a great variety in the manner and means of treatment—hence we have the same diversity of opinion, in regard to cure, as prevails in every other branch of the healing art! Take, for example, the single subject of stricture in the urethra. How many volumes have been written on the nature, causes, and methodus medendi of this complaint! Into what violent controversies have the advocates of simple and caustic bougies fallen! An octavo volume would hardly be sufficient to decribe the instruments which have been invented for opening out a free passage for the urine, when mechanically obstructed in the urethra.

The treatment of stricture has always hinged on two principles—mechanical dilatation, and chemical obliteration of the obstruction. The first principle is that which has been longest acted on, and is still preferred by a vast majority of surgeons. Sir Everard Home was the great advocate for the caustic in his day, and Mr. Andrews, the author of one of the works under notice, treads in his steps. Each principle has been put in practice in different ways. The argentum nitratum and the kali purum had and have their sworn advocates; while the principle of mechanical dilatation has given employment to the heads and hands of thousands, in the invention and construction of instruments. The wedge, or bougie, is chiefly used in this country—the dilator, as invented by Arnott, and improved and mounted with caustic by Ducamp, makes many converts in France.

Mr. Macilwain, whose small treatise lies before us, and which we consider to be the best manual which the student can take into his hands, decidedly prefers the successive introduction of bougies to the action of the dilator. His reasons are cogent. The mechanical obstruction cannot be *suddenly* removed by either plan—and that which is gradual and slow will be found the surest process. The dilator can be of no use where the stricture is incapable of admitting an instrument—and where it is capable, the dilatation can be effected by the introduction of a proper bougie. Mr. Macilwain prefers a silver catheter to all other instruments. "It admits of an exceedingly fine polish and uniformity of surface, which very much contribute to facility of introduction. Its lightness enables us to judge, with the greatest accuracy, of the force which the obstacle opposes to its progress, as well as that which we may employ to overcome it: further, we are assured of its entrance into the bladder by the urine escaping through it." Mr. M. has no hobby. He adverts to the inconvenience, pain, hæmorrhage, retention of urine, &c. which sometimes attend the caustic bougie; but he candidly admits that, "in relinquishing the argenti nitras, many have run into an opposite extreme." Impressed with the inconveniences, &c. above-mentioned, and believing that the kali purum "is an equally effectual, and a safer application," Mr. M. admits "that there are a few cases in which the use of the argenti nitras is to be preferred."

In considering the comparative efficacy of the different modes of practice, Mr. M. is guided by the facts which practice has presented to his observation. It is a great error, he thinks, to attempt the removal of all strictures by one method:—and it is equally erroneous to suppose that each case requires a different kind of treatment. The great objects in the management of stricture are—a restoration of the canal to its natural diameter—prevention of untoward symptoms during the cure—

and prevention of recurrence of the disease. The practitioner should always bear in mind "that stricture can only be overcome in a gradual manner." If the instrument be introduced too often, or the size too rapidly increased, the urethra will become so irritable, that a fortnight or more may elapse before we shall be able to proceed with the treatment. As long as the improvement is progressive, the surgeon and patient should be satisfied. Mr. M. thinks, and we agree with him, that it is sometimes impossible to prevent the recurrence of stricture. In commencing the treatment, Mr. M. very judiciously recommends attention to the state of the bowels, and to the diet. It is wonderful how bad secretions and too full a system of alimentation will keep up stricture, and obstruct the removal of it by the best mechanical means.

When Mr. M. is unable to get through the stricture, or to increase the size of the instrument progressively, he has then recourse to the kali purum, as employed by Whately. He has rarely met with any unfavourable consequences resulting from it. Increased tenderness for a few hours or a day may take place, and sometimes, though rarely, pain in micturition; but these are the worst consequences he has ever witnessed. Under ordinary circumstances, one or two applications of the kali will enable us to proceed with the treatment by means of the bougie.

But the cases in which Mr. M. particularly recommends the kali purum, are those attended with remarkable sensibility of the strictured part, the moment it is touched by the bougie, followed generally by a discharge of some blood. In all such cases, our author has adopted the usual means of allaying irritation, at the same time that he has employed the kali purum. The precise share which the latter may have therefore, in the reduction of morbid sensibility, he is not prepared to state. He has never used the argenti nitras, *in these cases*, and cannot speak practically as to its effects.

The cases to which Mr. M. limits the application of the lunar caustic are rare. The most remarkable feature in them is "extreme want of sensibility in the contracted portion." The rudest manipulations which are ever warrantable are unattended with pain, or succeeded by hæmorrhage. A very annoying symptom in this kind of stricture is the violent straining in making water—more distressing, however, than painful. In short, the character of this species is the very reverse of that for which our author has recommended the kali purum.

But, as our author acknowledges that he has not himself applied the argentum nitratum to the irritable strictures, we do not consider his limitation of the armed bougie to the insensible strictures as based on any very solid foundation. Indeed we have found the lunar caustic extremely useful in allaying this morbid irritability of the urethra, though the said irritability forms the grand objection, in Mr. Macilwain's mind, to the remedy.

In concluding this very short notice of Mr. Macilwain's little work, we have no hesitation in averring that it is the most judicious, concise, and ably written treatise on the subject which we have perused—and that it is most admirably adapted for putting the student and junior practitioner in possession of all the valuable information which has been accumulated on the subject of stricture.

Mr. Andrews' work is a second edition, the first having been printed 20 years ago, when the author resided at Madeira. It cannot be expected that we can take any lengthened notice of a second edition—and especially as a considerable portion of the work is occupied with criticisms on the writings and opinions of others. To the senior practitioner, this work will be more acceptable, on account of its critical examination of doctrines and practices emanating from men who enjoyed great popularity and fame in their day.

Mr. Andrews is a strenuous advocate for the lunar caustic in urethral strictures, and seems to have caught the mantle of Sir Everard Home, on this point of practice. He has given the "retort courteous" to Mr. Wadd, who appears to have handled our author's first edition somewhat roughly. Those surgeons who are inclined to the employment of lunar caustic, will find strong inducements to the practice in Mr. Andrews' book—and those who have taken up an unreasonable prejudice against the measure, as we are sure many have done, will probably alter their opinions, or at least modify them, on perusing this second edition, which contains numerous cases in illustration of the author's practice.

Among the objections to the use of lunar caustic in stricture of the urethra was urged the chance of the caustic slipping out of the bougie, and doing great injury to the canal. Professor Brande, however, has communicated to our author the discovery, that the muriatic salts of the urine *instantly* decompose the nitrate of silver, changing it into an inert and insoluble chloride of silver, by which transformation the caustic is, of course, disarmed of one of its greatest terrors. In the event, therefore, of the accident above alluded to, the patient has only to make water, and the decomposition of the caustic will be effected.

Upon the whole, we recommend the perusal of Mr. Andrews' work to all surgeons who have the important subject of urethral stricture to treat. We do not advocate the caustic so strongly as our author; but we believe—indeed we know, that an unreasonable dread, or at least an unmerited disuse of the remedy prevails among surgeons, which the statement of cases brought forward by Mr. A. is calculated to counteract.

